# Recurrent Acute Nonrheumatic Streptococcal Myocarditis Mimicking STEMI in a Young Adult

**DOI:** 10.1155/2014/964038

**Published:** 2014-05-22

**Authors:** Amanda Chikly, Ronen Durst, Chaim Lotan, Shmuel Chen

**Affiliations:** ^1^Department of Internal Medicine, Hebrew University, Hadassah Ein Kerem Medical Center, P.O. Box 12000, 91120 Jerusalem, Israel; ^2^Department of Cardiology, Hebrew University, Hadassah Ein Kerem Medical Center, P.O. Box 12000, 91120 Jerusalem, Israel

## Abstract

Myocarditis consists of an inflammation of the cardiac muscle, definitively diagnosed by endomyocardial biopsy. The causal agents are primarily infectious: in developed countries, viruses appear to be the main cause, whereas in developing countries rheumatic carditis, Chagas disease, and HIV are frequent causes. Furthermore, myocarditis can be indirectly induced by an infectious agent and occurs following a latency period during which antibodies are created. Typically, myocarditis observed in rheumatic fever related to group A streptococcal (GAS) infection occurs after 2- to 3-week period of latency. In other instances, myocarditis can occur within few days following a streptococcal infection; thus, it does not fit the criteria for rheumatic fever. Myocarditis classically presents as acute heart failure, and can also be manifested by tachyarrhythmia or chest pain. Likewise, GAS-related myocarditis reportedly mimics myocardial infarction (MI) with typical chest pain, electrocardiograph changes, and troponin elevation. Here we describe a case of recurrent myocarditis, 5 years apart, with clinical presentation imitating an acute MI in an otherwise healthy 37-year-old man. Both episodes occurred 3 days after GAS pharyngitis and resolved quickly following medical treatment.

## 1. Introduction

Myocarditis is an inflammatory disease of the cardiac muscle associated with both infectious and noninfectious diseases. Among the infectious etiologies, viruses are the most frequent pathogens, particularly in developed countries, while in the developing world rheumatic carditis,* Trypanosoma cruzi,* and bacterial infections such as diphtheria still contribute to the global burden of the disease [1]. The most common viruses involved are enterovirus (especially Coxsackie virus), adenovirus, and parvovirus-B19 [1, 2]. In 1947, Gore and Saphir described 12 cases of myocarditis in the setting of acute streptococcal tonsillitis that did not fit the revised Jones criteria for acute rheumatic fever (i.e., not occurring after the 2- to 3-week classical delay for rheumatic fever) [3]. They therefore titled this form of the disease “nonrheumatic myocarditis.” Subsequently, numerous cases of nonrheumatic myocarditis have been reported in the literature. The clinical manifestations are variable: it classically presents as an acute heart failure and can also be manifested with arrhythmia (tachycardia or bradycardia) or chest pain mimicking MI, especially in young patients, accompanied with focal ST elevations on the electrocardiogram and elevated cardiac biomarkers. A subset of patients with myocarditis will progress to chronic inflammatory and dilated cardiomyopathy, but most patients recover spontaneously without sequelae. Specifically, most patients with an acute MI-like syndrome spontaneously recover within hours to several days [2]. Nevertheless, acute postinfectious myocarditis is principally a monophasic inflammatory disorder. Cases of repetitive or recurrent myocardial inflammation after an initial episode of myocarditis, although reported, are extremely rare and usually occur shortly (weeks to months) following recovery from the first episode [4, 5]. No description of GAS-related nonrheumatic myocarditis recurrence has yet been reported in the literature. Here we present a case of a 37-year-old man who presented twice—five years apart—with myocarditis mimicking ST elevation myocardial infarction (STEMI) a few days after he was diagnosed with GAS pharyngitis.

## 2. Case Report

**Figure 1:**
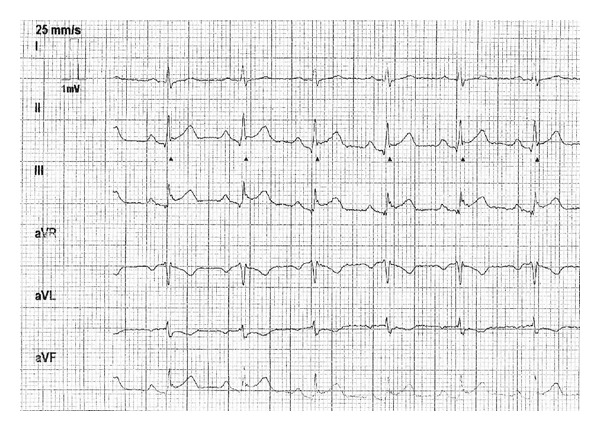
ECG on admission showing ST elevation in leads II, III, and aVF as well as slight reciprocal changes in leads I and AVL.

**Figure 2:**
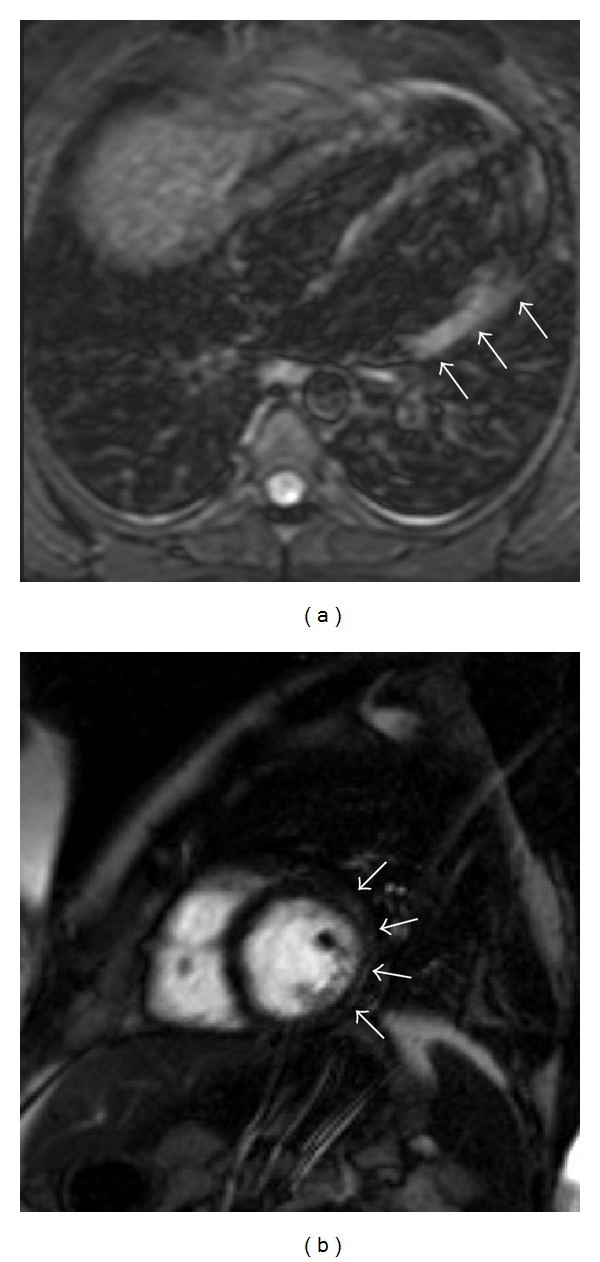
MRI finding of the patient. (a) Short TI inversion recovery (STIR) 4-chamber view showing hyperintense signal of the lateral inferior wall (arrows) suggesting focal edema. (b) Delayed enhancement of midmyocardial short axis showing hyperintense signal on the lateral inferior wall as well as delayed enhancement of the midepicardium and subepicardium (arrows).

A 37-year-old active healthy male with no history of smoking or drinking presented to the emergency department with chest pain. Five years earlier, he presented to the emergency department with chest pain radiating to the left shoulder and arm accompanied by shortness of breath. This occurred three days after he was diagnosed with group A streptococcal pharyngitis and he was treated with penicillin. Electrocardiogram demonstrated ST elevation in the inferior leads and troponin as well as CPK levels were elevated. The assumed diagnosis was STEMI and he was urgently catheterized. Catheterization indicated normal coronaries. Echocardiography demonstrated normal left ventricle function with inferior wall hypokinesia. Inflammatory markers (ESR and CRP) were elevated and he was diagnosed with myocarditis. He was treated with antibiotics and discharged home. During follow-up, ECG and echocardiography were normalized. Three days before the current admission, he presented with a pharyngeal infection. Throat swab for GAS was positive and he was treated with penicillin. On admission, he had a regular pulse of 96/min, blood pressure of 128/96 mm Hg, temperature of 36.8°, and 97% saturation while breathing room air. Physical examination was unremarkable, heart sounds were normal, there were no signs of heart failure, and there was no rash. Initial ECG showed sinus rhythm with ST elevation in leads II, III, and aVF and slight reciprocal changes in the lateral leads (I and aVL) consistent with inferior wall infarction (Figure 1). Echocardiogram supported the diagnosis of inferior STEMI, demonstrating an inferior wall akinesia. Urgent coronary angiography was performed, which demonstrated normal coronary arteries with no calcifications or stenosis. A few hours after presentation to the emergency room, the pain began to subside. Subsequent blood tests revealed troponin T (high sensitivity) level of 1.5 ng/mL (normal range: <0.04 ng/mL), CPK level of 1129 units/L (normal range: 26–192 units/L), CRP level of 19.6 Mg% (normal range: <0.5 Mg%), hemoglobin level of 13.1 (normal range: 14–18 GR%), and white blood cell count of 14 (normal range: 4–10 × 10^9^/L), with 87% neutrophils, 6.2% lymphocytes, 0.1% eosinophils, and 6.6% monocytes. Platelets 142 (normal range 140–400 × 10^9^/L). INR 1.1 (normal range 1–1.4). Antistreptolysin positive. Antinuclear antibodies—ANA negative and serum complement—C3 C4 levels were normal. Anticardiolipin antibodies, IgM and IgG, were negative. Viral serology for parvovirus also tested negative. MRI demonstrated hyperintense areas in the inferolateral wall in short TI inversion recovery (STIR), as well as delayed enhancement of the mid- and epicardial portions of the corresponding segments (Figure 2). These findings support the diagnosis of myocarditis. He was treated for the pharyngeal infection and discharged in good health. ECG and echocardiography upon follow-up were normalized.

## 3. Discussion

Myocarditis associated with group A streptococcus pharyngitis is a well-recognized condition and should be suspected in young patients with chest pain and ECG changes suggestive of MI, when occurring a few days after the pharyngeal infection. Yet, recurrence of this condition has not been reported thus far. Here we present a patient who had two episodes of typical ischemic chest pain with ST-segment elevation in the inferior wall and elevated troponin T, as well as inferior wall hypokinesia in echocardiography compatible with STEMI, while angiography concurrently demonstrated normal coronary arteries. These two events occurred five years apart with complete clinical, electro-, and echocardio-graphic resolution a few days after the onset.

The differential diagnosis in this patient includes ischemic event and myocarditis. The first is less likely to be the cause due to low pretest probability for ischemia, clinical picture, elevated inflammatory markers, and negative coronary angiography. Nevertheless, microvascular disease (see below) as well as coronary spasm cannot be ruled out. A more likely diagnosis is myocarditis which can be idiopathic/viral induced, GAS-related, part of systemic inflammatory disease (i.e., collagen disease), or eosinophilic myocarditis hypersensitivity induced by treatment with penicillin. The last is less reasonable because the patient's blood count demonstrated no eosinophilia (0.1 × 10^9^/L; normal range: 0.04–0.4 × 10^9^/L) and his clinical presentation, as well as the ECG changes, resolved under this treatment; the patient continued the treatment 7 days after the presentation (to complete 10 days of antibiotic treatment). Regarding systemic inflammatory disease, this patient had no other complaints (rash, arthritis, etc.) and excluding the two events previously depicted, he was a healthy individual. Moreover, immunologic serology tested negative as mentioned. Idiopathic event cannot be ruled out despite negative serology; yet, the temporal relation between the GAS pharyngitis and the myocarditis (twice) suggests an association rather than coincidence.

Myocarditis secondary to GAS infection has been described mainly as part of acute rheumatic fever. In this case, myocarditis occurs 2 to 4 weeks after the bacterial infection. In contrast, several cases indicate myocarditis occurred during or a few days after the streptococcal infection [6–8]. As mentioned above, this entity first described by Gore and Saphir in 1947 has been called nonrheumatic myocarditis [3]. This type of myocarditis typically occurred in young patients, mostly males, within a few days after onset of sore throat, with nonpleuritic chest pain and occasional fever. Additionally, anatomically localized ST-segment elevations on electrocardiography and elevated cardiac biomarkers were present in all cases. Coronary angiography, when performed, demonstrated normal arteries. All patients recovered after a relatively short period of time (days to weeks) with no sequelae. Acute MI mimicry is a nonclassical but well-known presentation of myocarditis, regardless of cause. Furthermore, the possibility of myocarditis should be raised in any case of clinical presentations of an acute MI in a patient with a normal angiogram. Sarda et al. investigated 45 patients admitted for suspected acute coronary syndrome, all who had normal coronary angiograms and underwent heart scintigraphy (indium-111-antimyosin antibody (AMA) and dual isotope rest AMA/rest thallium 201 imaging). 38% of the patients had diffuse myocarditis and 40% had a scintigraphic pattern of heterogeneous or focal myocarditis [9]. In another study, endomyocardial biopsy was performed on 12 patients with suspicion of acute MI and normal coronary angiography. The results showed obvious myocarditis according to the Dallas criteria in 6 patients, 1 with borderline findings, and in only one out of 12 patients, no inflammation was detectable [10]. It was suggested that such a presentation of myocarditis can be a result of coronary microvascular dysfunction, with heightened sensitivity of the coronary microvascularisation to vasoconstrictor stimuli and limited microvascular vasodilator capacity [11].

Recurrent myocarditis has been reported previously, secondary to different causes or without an identified cause, but not subsequent to GAS pharyngitis. Xu et al. recently reported two cases of recurrent myocarditis occurring in young men, a few years apart, with a presentation of acute MI, each time with normal coronary arteries demonstrated upon angiography. The causal agent was not found, but the events occurred following episodes of gastroenteritis or upper respiratory tract infection [5]. Other cases have been reported after streptococcal pneumonia vaccine [12], after a bout of the flu [4], or concomitant to Coxsackie's virus infection [13]. In the case presented above, GAS infection, as proven by a throat swab at the time of the pharyngeal infection, is suggested to be the trigger for the subsequent myocarditis. However, the long period between the two events (5 years) makes it unlikely that the events are connected, rather these are two unrelated events, most probably due to predisposition of the patient.

## 4. Conclusion

In conclusion, myocarditis presenting as an acute MI following GAS tonsillitis may reoccur, even after a few years following the first event, with a complete spontaneous remission after both episodes. The clinical picture can be explained by a focal microvascular vasoconstriction as a reaction to the local inflammatory stimulus. Consequently, in patients with predisposition, exposure to the infectious agent might provoke this reaction repeatedly.
